# Optimal design of cluster randomised trials with continuous recruitment and prospective baseline period

**DOI:** 10.1177/1740774520976564

**Published:** 2021-03-08

**Authors:** Richard Hooper, Andrew J Copas

**Affiliations:** 1 Centre for Clinical Trials & Methodology, Institute of Population Health Sciences, Queen Mary University of London, London, UK; 2 MRC Clinical Trials Unit at University College London, London, UK

**Keywords:** Efficient design, group randomised trials, power, sample size

## Abstract

**Background:**

Cluster randomised trials, like individually randomised trials, may benefit from a baseline period of data collection. We consider trials in which clusters prospectively recruit or identify participants as a continuous process over a given calendar period, and ask whether and for how long investigators should collect baseline data as part of the trial, in order to maximise precision.

**Methods:**

We show how to calculate and plot the variance of the treatment effect estimator for different lengths of baseline period in a range of scenarios, and offer general advice.

**Results:**

In some circumstances it is optimal not to include a baseline, while in others there is an optimal duration for the baseline. All other things being equal, the circumstances where it is preferable not to include a baseline period are those with a smaller recruitment rate, smaller intracluster correlation, greater decay in the intracluster correlation over time, or wider transition period between recruitment under control and intervention conditions.

**Conclusion:**

The variance of the treatment effect estimator can be calculated numerically, and plotted against the duration of baseline to inform design. It would be of interest to extend these investigations to cluster randomised trial designs with more than two randomised sequences of control and intervention condition, including stepped wedge designs.

## Introduction

In a cluster randomised trial participants from the same ‘cluster’ (e.g. patients attending the same general practice, or the residents of a predefined geographical region) are randomised to receive the same intervention.^1^ As in an individually randomised trial, there may be an advantage in assessing outcomes in clusters at ‘baseline’– pre-randomisation – in order to control for cluster differences and thereby increase precision.^2,3^ With some statistical modelling, we ought to be able to quantify whether it is worth devoting effort to collecting baseline data if this means we lose an opportunity to collect follow-up data.

The case of a cluster randomised trial with two repeated cross-sections – one baseline and one ‘endline’– has been presented previously.^4^ In this article, we study the case of a trial in which clusters prospectively recruit or identify participants as a continuous process over a given calendar period. We assume that the control is routine care, and that clusters are allocated 1:1 to intervention and control arms. In designing such an evaluation, an investigator might reasonably ask whether they should introduce the intervention to intervention clusters straightaway, or schedule a period of baseline data collection first, and in the latter case how long the baseline should be.

**Figure 1.**
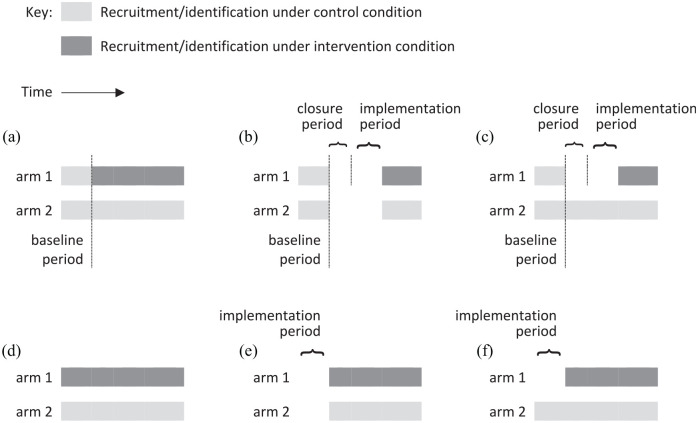
Designs for two-arm, cluster randomised trials with continuous recruitment and a prospective baseline. (a) No transition period; (b) recruitment/identification suspended in both arms during a transition period; (c) recruitment/identification suspended in the intervention arm, but not the control arm, during a transition period; (d)–(f) as for (a)–(c), but the intervention arm begins the trial in the intervention condition, rather than beginning in the control condition and crossing over to the intervention.

This ‘baseline’ is a period of *prospective* data collection (of duration set by the investigator) during which participants from all clusters are receiving routine care. This is followed (for the remaining time available) by a more conventional trial scheme in which half of the clusters cross over to the intervention condition and a new series of participants is recruited from each cluster (Figure 1).

### Motivating examples

Project Masihambisane was a cluster randomised trial of a peer mentoring programme to improve outcomes for pregnant mothers living with HIV in KwaZulu-Natal, South Africa.^5,6^ Eight antenatal clinics took part, and were randomised to the intervention or to routine care (four clinics in each arm). The peer mentoring programme was then introduced at intervention clinics. Eligible women (less than 34 weeks pregnant and HIV seropositive) were subsequently enrolled in clinic waiting rooms over a period from July 2008 to April 2010. The primary outcome was a composite score made up of indices of maternal and child health and well-being. The investigators calculated that they would need to recruit 1200 women in total (150 per clinic) to achieve adequate statistical power.

A slightly different design was adopted by the PRISMA trial of a multifaceted intervention to reduce perinatal morbidity among women with a previous caesarean delivery in Québec.^7^ In this trial, 40 hospitals were randomised 1:1 to either intervention or routine care. The trial used routinely collected data at the participating hospitals. All eligible women delivering at one of the hospitals during a specified period were included. Rather than implementing the intervention straightaway in the intervention arm, however, the investigators specified that there should be a 1-year pre-intervention (baseline) period of recruitment in both arms, followed by a 5-month transition period (during which the intervention was to be introduced to 20 of the hospitals), followed by a 2-year post-intervention period of recruitment in both arms. The primary outcome was perinatal mortality, and the investigators calculated that 40 clusters would achieve adequate statistical power, on the assumption that participants were identified at a rate of 184 per hospital per year.

The question arises whether in Project Masihambisane it might have been more efficient to schedule a baseline period, and, in both examples, how long this baseline period ought to be, relative to the overall period of recruitment. In this article, we show how theoretical trial performance can be plotted against cross-over time to help make this decision. We also offer some general advice on the design of this kind of trial.

## Methods

### Statistical model

We restrict attention to trials with continuous outcome measures. Suppose that participant $i=1,\ldots ,m_{j}$ in cluster j=1,…,2J is recruited or identified at time $t_{\mathrm{ij}}$ . We assume that each participant has the primary outcome assessed once, at a fixed time following recruitment or identification. We also assume each participant can be identified unambiguously as being in either the control or the intervention condition (more on this to follow). Suppose we cross *J* clusters over to the intervention at time $t^{*}$ .

Our model for outcome $Y_{\mathrm{ij}}$ of participant *i* in cluster *j* is

$$Y_{\mathrm{ij}}=T(t_{\mathrm{ij}})+\delta_{\mathrm{ij}}\theta +\varepsilon_{\mathrm{ij}}$$

where $\delta_{\mathrm{ij}}$ is 1 if cluster *j* is in the intervention arm and $t_{\mathrm{ij}}\geq t^{*}$ , and 0 otherwise. The parameter θ is the treatment effect we want to estimate.

The function *T* is the fixed effect of time on outcome. Since we think of time as continuous it may be appropriate to assume that *T* is a continuous function: in this article we consider polynomial functions. Note that when it comes to the analysis, the form of the time effect may not be of great interest. Indeed, in the analysis of trials of this kind T is often treated as a crude step function: piecewise constant, but with a discontinuity at precisely the cross-over point $t^{*}$ . Although it might seem odd that the true, underlying time trend should depend on the choice of design (i.e. on the choice of $t^{*}$ ), there could be situations where a control group experiences a step change in outcomes at exactly the same time as the intervention group, either coincidentally or because control clusters are aware of the cross-over, and this would require adjustment. We consider time effects with and without a discontinuity at $t^{*}$ .

It is common when modelling longitudinal cluster randomised trials to allow the intracluster correlation (the correlation between outcomes of two individuals from the same cluster) to depend on the time elapsed between sampling the individuals.^8,9^ Here, we allow our correlation to decay continuously over time with the following parameterisation (where we assume that each $Y_{\mathrm{ij}}$ has the same variance)

$$\begin{array}{c} \mathrm{Corr}(Y_{i_{1}j},Y_{i_{2}j})=\rho \tau^{\left|t_{i_{1}j}-t_{i_{2}j}\right|}\\ \mathrm{Var}(Y_{\mathrm{ij}})=\sigma^{2} \end{array}$$

Time, we will assume without loss of generality, runs from 0 to 1 over the course of the trial, hence the correlation between the outcomes of two individuals from the same cluster sampled at either end of the recruitment period is τρ , while the correlation between the outcomes of two individuals from the same cluster sampled at the same time is ρ . If τ=1 , there is no decay in the intracluster correlation.

The general model outlined above represents, if you like, a kind of cluster randomised interrupted time series analysis, with each cluster randomised either to a condition in which routine care is interrupted at a predetermined time by the introduction of the intervention, or to a control condition.

The precision of the generalised least squares estimate of the treatment effect has a well-known expression when the analysis model is correctly specified. Formally, if we write outcomes $Y_{\mathrm{ik}}$ as a single column vector **Y**, and parameters for fixed effects (including time effects and treatment effect) as a column vector θ , and express the linear model above in matrix form

Y=Zθ+e,e~N(0,V)

then the variance of the generalised least squares estimator for θ is

(1) $$\mathrm{Var}(\hat{\theta })=(Z\prime V^{-1}Z{)}^{-1}$$

The results presented in this article were obtained by numerical matrix inversion in Stata (Stata Corporation, College Station, TX, USA). Code for calculating the variance of the treatment effect estimator under different designs is accessible from our GitHub repository (https://github.com/richard-hooper/CRT-continuous-recruitment-prospective-baseline).

### Model for recruitment or identification

Schematics for two-arm, longitudinal trial designs are illustrated in Figure 1. Although we are ultimately interested in situations where eligible participants present at a cluster as a random process in continuous time, we will simplify by imagining that eligible participants arrive at each cluster at regularly-spaced times 1/m,2/m…m/m , (where *m* is the arrival rate at each cluster, i.e. the cluster size).

It may be necessary to include a transition period in the intervention arm between the recruitment or identification of participants under the control condition and under the intervention condition. A transition period typically comprises two distinct periods: a ‘closure’ period long enough for all control participants to have had their outcomes assessed or else to have ‘left’ the cluster (i.e. no longer be exposed to interventions), and an ‘implementation’ period long enough to implement the intervention at the cluster and have it running at full strength (Figure 1(b)).^10^ To avoid bias, and to satisfy the assumption that each participant can be identified unambiguously as being in either the control or the intervention condition, outcomes from the transition period in the intervention arm should be excluded from the primary analysis.^10^

Unless outcomes are routinely collected, an investigator may prefer to suspend recruitment completely in the intervention arm during the transition period. Note that if the intervention arm is to begin the trial in the intervention condition, with no baseline period, then there is no need for a closure period: recruitment need only be delayed by the time it takes to implement the intervention in this case (Figure 1(e)). In studies where the closure period is appreciable this offers increased opportunity for data collection, and hence perhaps some statistical power advantage compared with the design in Figure 1(b).

What is less clear is whether we should continue recruitment in the control arm while the intervention arm is in transition (or, for routinely collected outcomes, whether to include control arm outcomes from this period in the analysis), as illustrated in Figures 1(c) and (f). This violates the principle of having concurrent intervention and control participants, but with some statistical modelling it could offer greater precision for estimating the treatment effect. We investigate designs with and without recruitment in the control arm during the transition period.

Under the model for recruitment described above (i.e. assuming that each of the J clusters in the intervention arm generates the same number of observed outcomes $Y_{\mathrm{ij}}$ with the same joint distribution, and similarly for each of the J clusters in the control arm) the variance in equation (1) will be proportional to $\sigma^{2}/J$ . This variance can be evaluated (as we will do in this article) for $\sigma^{2}=1$ and J=1 in order to obtain a multiplier that can be used to calculate the variance for any $\sigma^{2}$ and J . Note that in practice no cluster randomised trial would have J=1 (one cluster in each arm).

### Scenarios investigated

We plot the variance of the treatment effect estimator for different cross-over times, for all combinations of m=25,50,100,200 , ρ=0.001,0.005,0.01,0.05,0.1 , and τ=1.0,0.5,0.1 , with no transition period. We also illustrate what happens if there is a transition period (12.5%, 25%, 37.5%, or 50% of the total recruitment period available), for different τ , in the case m=100,ρ=0.05 , and we compare the trial performance with or without the inclusion of control data from the transition period.

We consider two kinds of fixed time effect: a cubic polynomial and a piecewise constant function with a discontinuity at the cross-over (step function). Note that as long as we include a piecewise constant time effect with a discontinuity at the cross-over in our model, then whatever other fixed effects of time we also include in the model (such as an additional linear trend or cubic polynomial), the variance of the treatment effect estimator will remain the same. This is a corollary of a more general invariance theorem proved by Grantham et al.,^11^ and follows because the pattern of control and intervention conditions in the intervention arm corresponds exactly to the piecewise constant time effect. Hence we label our findings in the latter case as being simply for a ‘discontinuous’ time effect.

To illustrate how the precise form of the time effect might influence our conclusions, we illustrate results for polynomial time effects with polynomial degrees 2 up to 6, alongside results for a discontinuous time effect, in the case m=100,ρ=0.05,τ=0.5 , with no transition period.

Finally, we apply our approach to the design of Project Masihambisane, one of the trials we introduced earlier as a motivating example.

## Results

**Figure 2.**
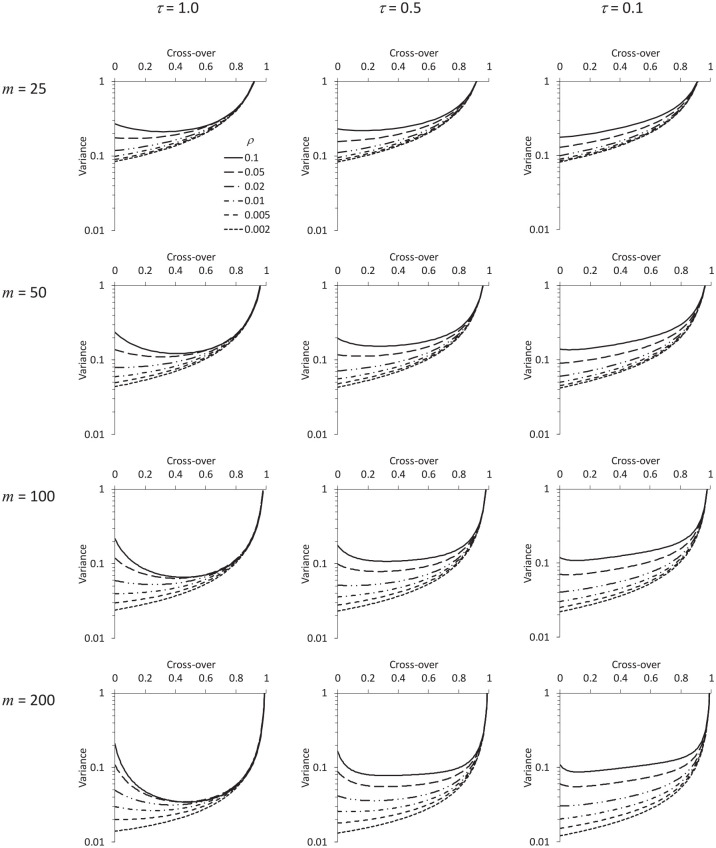
Variance of the treatment effect estimator according to the number of participants recruited in each cluster (*m*), the timing of cross-over in the intervention arm, the intracluster correlation for two participants sampled from the same cluster at the same time (*ρ*), and the factor by which this intracluster correlation is reduced for two participants sampled from the same cluster at opposite ends of the trial period (*τ*). There is no transition period, and the time effect is assumed to be a discontinuous at the cross-over. The variance in a given application is the value shown on the axis multiplied by $\sigma^{2}/J$ , where $\sigma^{2}$ is the variance of the outcome and J is the number of clusters in each arm.

**Figure 3.**
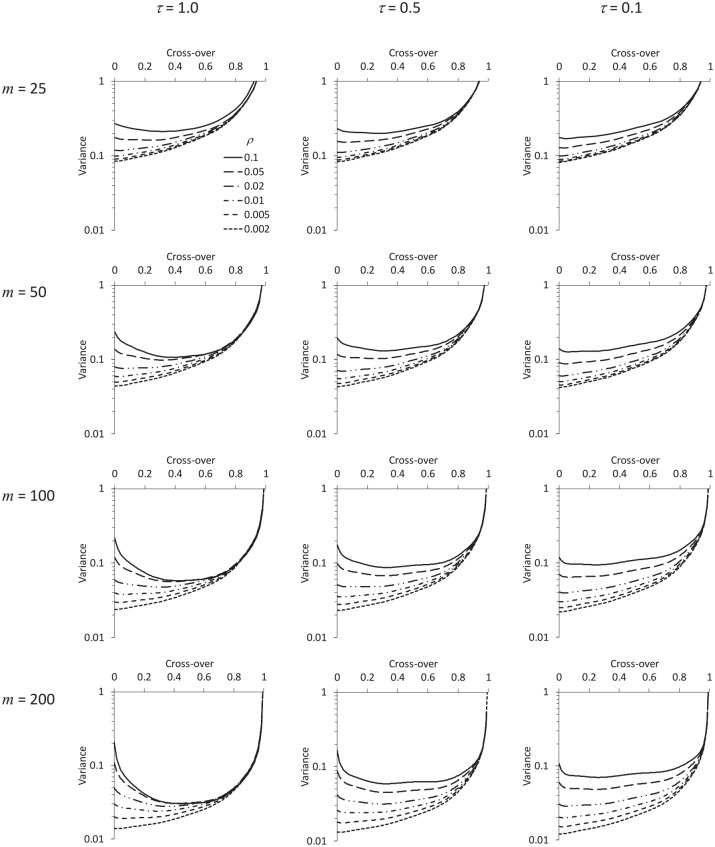
As Figure 2, but the time effect is assumed to be cubic polynomial.

**Figure 4.**
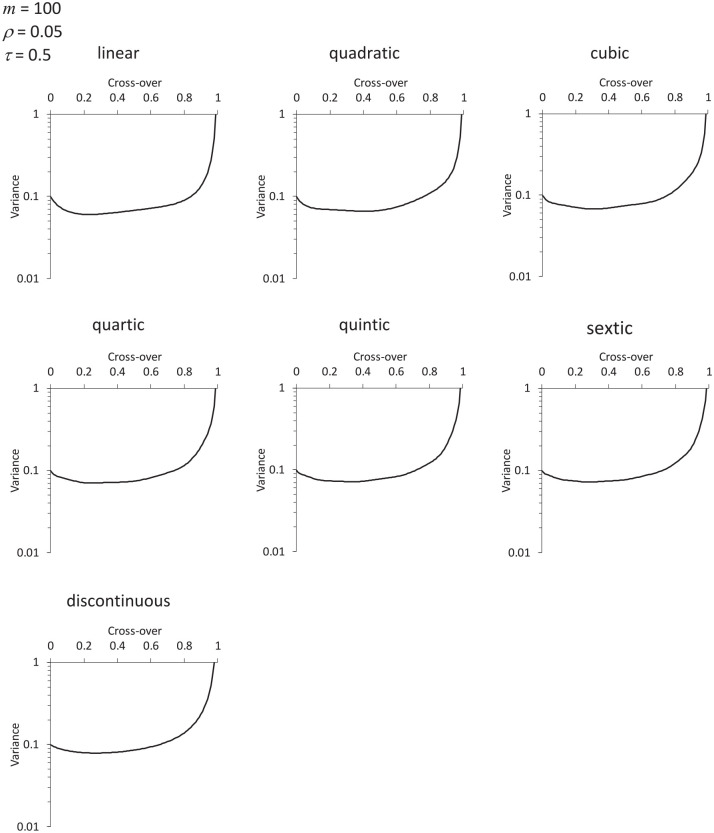
Results in the case m=100,ρ=0.05,τ=0.5 , with no transition period, according to the degree of the polynomial assumed for the time effect. At the bottom are the results when the time effect is discontinuous at the cross-over.

Figures 2 and 3 show how the variance of the treatment effect estimator depends on the cross-over time, for different combinations of m , ρ and τ , with no transition period. Figure 2 assumes a discontinuous effect of time, and Figure 3 assumes a cubic polynomial effect. The figures are remarkably similar. Indeed, Figure 4 shows how (in the particular example m=100,ρ=0.05,τ=0.5 ) as we increase the degree of the polynomial effect of time from linear, to quadratic, cubic, and through to sextic, the variance curve approaches the form for a discontinuous time function, as if asymptotically. This suggests that some generalisable conclusions may be drawn regarding optimal design choices, irrespective of the form of the time effect.

Observe, also, how the shape of each curve in Figures 2 and 3 depends principally on τ and mρ : that is, for given τ , if m doubles and ρ halves then the shape of each curve remains roughly the same. This suggests that we should be able to choose an optimal design based only on τ and mρ .

The figures reveal that in some scenarios the optimal design is to start the intervention arm in the intervention condition (i.e. to have no baseline period), while in others there is an optimal duration for the baseline. The optimal proportion of data collection effort dedicated to the baseline is anything up to one-half, but no more (just as seen in the case of a trial with two repeated cross-sections – one baseline and one endline).^4,12^

The graph may be used to identify the optimum in a given scenario, but we observe that as a practical rule of thumb, for given ρ and τ , investigators would not go far wrong by choosing from the two specific options of having no baseline period at all, or having a baseline period taking up half of the trial period, whichever leads to the smaller variance. In every case in Figure 2, this strategy is close to optimal in terms of the variance achieved. As a rough rule of thumb, then, when there is no transition period, a baseline period is unnecessary for τ=1 when mρ is around 1 or smaller, for τ=0.5 when mρ is around 2 or smaller, and for τ=0.1 when mρ is around 5 or smaller.

In practice, of course, we may not be sure of the values of ρ and τ . Suppose we want to choose a duration for the baseline that minimises the maximum variance over a range of plausible ρ or τ . Figure 2 illustrates how, for given ρ , the value of τ that leads to maximum variance depends on the length of the baseline. If our goal was to minimise the maximum variance over a wide range of τ we might end up concluding, on consulting plots like those in Figure 2, that the best choice of baseline was something intermediate between no baseline and a baseline taking up half the trial period.

**Figure 5.**
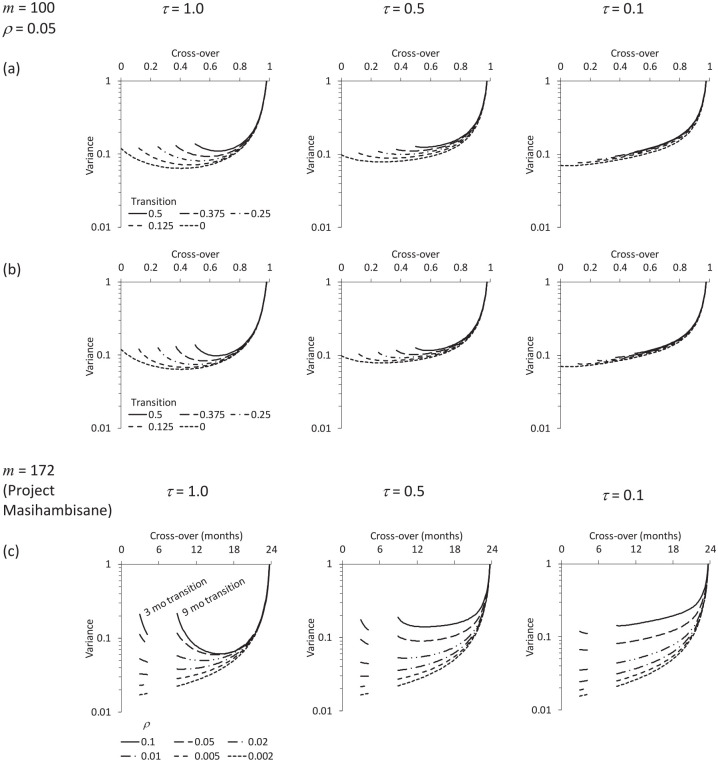
Effect of a transition period on the variance of the treatment effect estimator. Cross-over is the time at which recruitment/identification under the intervention condition begins in the intervention arm. The time effect is assumed to be discontinuous at the cross-over. (a) and (b) m=100,ρ=0.05 with recruitment/identification in the control arm (a) suspended or (b) continued during the transition period; (c) the example of Project Masihambisane, running for 24 months with a 9-month transition period (or a 3-month transition period if the intervention is implemented straight away, with no baseline).

**Figure 6.**
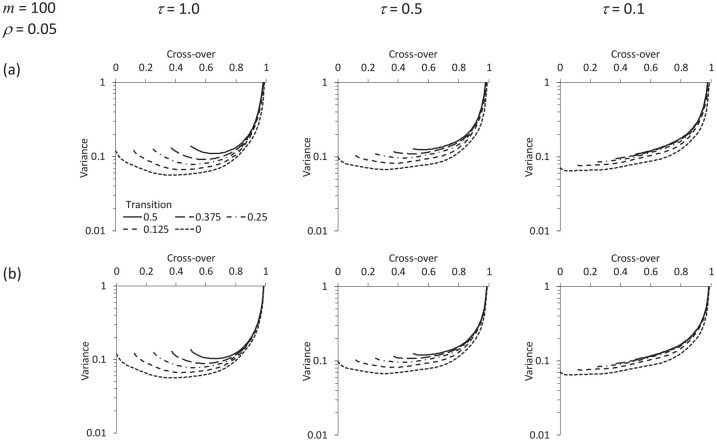
As Figure 5, but the time effect is assumed to be a cubic polynomial. (a) and (b) m=100,ρ=0.05 with recruitment/identification in the control arm (a) suspended or (b) continued during the transition period.

Figures 5 and 6 illustrate what happens to the variance curve when there is a transition period. A transition period effectively reduces the number of participants per cluster and also (if τ<1 ) the correlation between outcomes before and after introduction of the intervention in a cluster. Still, for given m , ρ and τ the overall shape of the curve seems to be changed little unless τ is small and the transition period is wide: the effect of including a transition period is principally to compress the curve within a narrower window of possible cross-over times, and to increase the variance.

Comparing Figure 5(a) and (b) (or Figure 6(a) and (b)) shows that including control data from the transition period has only a modest impact on the precision of the estimate. In the example that follows, we assume data are excluded from both the control and intervention arms over the transition period.

### Example

Project Masihambisane, described earlier, ran over a total of 21 months, and the sample size calculation for the trial assumed that 150 participants would be recruited at each cluster in this time (i.e. 86 per cluster per year). The trial was designed to achieve 80% power to detect a standardised mean difference in outcomes of 0.25.^5,6^ No details were given of assumptions regarding the intracluster correlation.

Peer mentors who delivered the Project Masihambisane intervention worked fulltime in the participating antenatal clinics. The intervention is conducted over four antenatal visits, and clinics have contact with participating women for 6 weeks after birth, so in a design that is to include a baseline period of recruitment, where all clinics are delivering routine care, it would be prudent to follow this with a closure period of, say, 6 months to allow participants recruited under the routine care condition to have passed through the system. Peer mentors also require initial training, so we need to allow for an implementation period of, say, 3 months. Let us suppose that in Project Masihambisane this training period preceded the 21 months of data collection, so that the total available time for running the trial, including implementation, was 24 months, with m in this case being 172.

A design with a baseline period would require an overall transition period of 9 months (6 months closure and 3 months implementation). Figure 5(c) shows plots of the variance of the treatment effect estimator, for different ρ and τ . The plots also show where the respective variance curves would start if the transition period was just the 3-month implementation period. The *starting* point of the latter curve (at 3 months) is important because it shows how a design with no baseline period would perform.

Suppose, for example, that ρ=0.05 and τ=1 . Then the optimal design is to cross over at around 15 months, with a variance of $0.060\sigma^{2}/J$ . Note that this is not far from the variance for a more symmetrical design that crosses halfway between 9 and 24 months – that is, a design with a 7.5-month baseline period, followed by a 9-month transition period, followed by a 7.5-month follow-up period – which works out as $0.061\sigma^{2}/J$ . In order to detect an effect size of $\theta^{*}$ with power (1−β) at significance level α we need

$$J\geq 0.060{\left(\frac{\sigma }{\theta^{*}}\right)}^{2}{\left(z_{1-\alpha /2}+z_{1-\beta }\right)}^{2}$$

where $z_{p}$ is the 100*p*th centile of a standard normal distribution. So, to detect a standardised mean difference of 0.25 with 80% power at the 5% significance level requires eight clusters per arm using the formula above, though due to problems arising from small numbers of clusters it may be wise to add one or more clusters per arm.^13,14^

Now suppose, alternatively, that ρ=0.02 and τ=0.5 . Then the optimal design with a baseline period is to cross over at around 10 months, giving a variance of $0.052\sigma^{2}/J$ . But note what happens if we dispense with the baseline period altogether and begin the trial after an implementation period of just 3 months: this results in an even smaller variance, of $0.046\sigma^{2}/J$ . To detect a standardised mean difference of 0.25 with 80% power at the 5% significance level using the latter design requires six clusters per arm using the formula above.

## Discussion

In considering the benefits of a prospective baseline period in a cluster randomised trial with continuous recruitment/identification of participants over a fixed calendar period, we find that in some circumstances it is optimal not to include a baseline, while in others there is an optimal duration for the baseline. We also note that in most circumstances investigators could achieve close to optimal precision either with a design that has no baseline or with one that divides the available time in half – a ‘none or half’ approach – though they would still need to evaluate the performance of both these options, and it may be just as easy (and more informative) to plot performance over all possible cross-over times.

All other things being equal, the circumstances where it is preferable *not* to include a baseline period are those with a smaller recruitment rate, smaller intracluster correlation, greater decay in the intracluster correlation over time, or wider transition period (particularly if this includes an appreciable closure period). If there is a transition period between recruiting or identifying participants under the control and intervention conditions in the intervention arm, then there may only be a modest benefit to having data available from the control group during this transition period.

Our conclusions seem to be robust to the form of the underlying time effect, so when designing a trial there may be little point in trying to predict exactly what the form will be. In practice there will be an advantage to adjusting appropriately for the actual time effect at analysis, if we know its functional form, since this will improve precision and is the basis on which we calculated sample size. Nevertheless, adjusting simply for a piecewise constant effect of time with a discontinuity at cross-over is still an attractive approach to analysis since it should give an estimate of the treatment effect that is unbiased, at least if the pattern of recruitment is the same in control and intervention clusters.

The calculation of the variance of the treatment effect estimator in a generalised least squares framework, and hence of required sample size in different scenarios, is achieved with numerical matrix inversion, and needs some coding. We have not been able to derive analytical expressions for required sample size, in general. However, if the intracluster correlation is uniform over time ( τ=1 in our model), then sample size can be calculated using methods for cluster randomised trials with repeated cross-sections and no decay in the intracluster correlation.^4,15,16^ If τ=1 and there is no baseline period, then the sample size calculation problem reduces to that for a straightforward, parallel groups cluster randomised trial.^17,18^

We assumed a particular parametric form for the decay in the intracluster correlation to help us understand the more general impact of this kind of decay on optimal design. Other models for the intracluster correlation could, of course, be investigated. When designing a trial in practice, an investigator will want reassurance that methods exist for analysing the data that can accommodate suitable intracluster correlation structures: a decaying correlation such as we have assumed can be specified as part of a mixed regression model in SAS PROC MIXED (SAS, Cary NC, USA), with the nlme package for R,^19^ or in ASReml for R (VSNi, Hemel Hempstead, UK), for example. More software solutions may become available over time.

We simplified considerably in assuming that eligible participants present at regular, fixed intervals rather than as a random continuous-time process, but assuming that the arrival rate is constant over time we would expect arrival times in a sample to become increasingly uniformly spread as the rate increases. Simulation studies that have investigated the impact of unevenly spaced arrival times on precision of the treatment effect estimator in the context of stepped wedge designs suggest that this impact is small.^9^

We have limited our attention in this article to two-arm designs. It would be of interest to extend these investigations to cluster randomised trial designs with more than two randomised sequences of control and intervention condition, including stepped wedge designs.
